# Auditory Resting-State Network Connectivity in Tinnitus: A Functional MRI Study

**DOI:** 10.1371/journal.pone.0036222

**Published:** 2012-05-04

**Authors:** Audrey Maudoux, Philippe Lefebvre, Jean-Evrard Cabay, Athena Demertzi, Audrey Vanhaudenhuyse, Steven Laureys, Andrea Soddu

**Affiliations:** 1 Coma Science Group, Cyclotron Research Centre, University of Liège, Liège, Belgium; 2 OtoRhinoLaryngology Head and Neck Surgery Department, University of Liège, Liège, Belgium; 3 Radiology Department, CHU Sart Tilman Hospital, University of Liège, Liège, Belgium; 4 Neurology Department, CHU Sart Tilman Hospital, University of Liège, Liège, Belgium; Centre Hospitalier Universitaire Vaudois Lausanne - CHUV, UNIL, Switzerland

## Abstract

The underlying functional neuroanatomy of tinnitus remains poorly understood. Few studies have focused on functional cerebral connectivity changes in tinnitus patients. The aim of this study was to test if functional MRI “resting-state” connectivity patterns in auditory network differ between tinnitus patients and normal controls. Thirteen chronic tinnitus subjects and fifteen age-matched healthy controls were studied on a 3 tesla MRI. Connectivity was investigated using independent component analysis and an automated component selection approach taking into account the spatial and temporal properties of each component. Connectivity in extra-auditory regions such as brainstem, basal ganglia/NAc, cerebellum, parahippocampal, right prefrontal, parietal, and sensorimotor areas was found to be increased in tinnitus subjects. The right primary auditory cortex, left prefrontal, left fusiform gyrus, and bilateral occipital regions showed a decreased connectivity in tinnitus. These results show that there is a modification of cortical and subcortical functional connectivity in tinnitus encompassing attentional, mnemonic, and emotional networks. Our data corroborate the hypothesized implication of non-auditory regions in tinnitus physiopathology and suggest that various regions of the brain seem involved in the persistent awareness of the phenomenon as well as in the development of the associated distress leading to disabling chronic tinnitus.

## Introduction

Tinnitus is defined as a perception of sound in the absence of any external auditory stimuli [1]. It is sometimes referred to as ‘phantom’ auditory experience. About 15% of the population is affected by chronic tinnitus and tinnitus severely affects quality of life of 1 to 3% of the population [2]. Despite its high prevalence, there is little consensus regarding the neuropathological origin of tinnitus. The prevailing opinion is that tinnitus is a perceptual consequence of altered patterns of intrinsic neural activity generated along the central auditory pathway following damage to peripheral auditory structures [2]. While the loss of afferent input to the central auditory system can initiate tinnitus, thereafter, central mechanisms are thought to play an important role in its maintenance [3]. That surgical section of the eight cranial nerve in tinnitus patients is not successful in suppressing tinnitus in 38 to 85% of the cases further supports this hypothesis [4], [5]. A better characterization of central neural processing abnormalities in tinnitus can offer a better understanding of the physiopathology and may contribute to the development of therapeutic intervention procedures.

Few studies on tinnitus have assessed cerebral functional connectivity changes. Previous electrophysiological studies suggested evidence of modified connectivity in tinnitus subjects [6], [7], [8], [9]. However, the use of magnetoencephalography (MEG) or electroencephalography (EEG), while providing high temporal resolution, is known to have a poor anatomical resolution making difficult precise interpretation on the exact location of the source of the signal. One way to overcome this limitation is to use a functional brain imaging technique which, even if more limited concerning temporal resolution, has better structural resolution (e.g. functional MRI).

Since it has been shown that correlation of low frequency fluctuations (0.01–0.05 Hz) of resting BOLD activity reflect functional connectivity [10], an increased focus has been directed to functional MRI studies of the brain's baseline activity (i.e., “resting state” acquisitions) [11]. Indeed, these fluctuations are shown to be coherent across widely separated (although functionally related) brain regions, constituting “resting state networks” [12], [13]. Past studies in healthy volunteers showed that it is possible to identify consistent resting-state networks that have a functional relevance. “Default” network or networks involved in visual, motor, language, and auditory processing can be consistently found in healthy subjects [14], [15] and can be separated from each other from a single resting-fMRI dataset using their distinct temporal characteristics. Maps of spontaneous network correlations have been proposed to provide tools for the understanding of clinical conditions. fMRI resting-state paradigms have, for example, been applied to the study of hypnosis [16], anesthesia [17] and various neurological disorders including dementia [18], [19], depression [20] disorder of consciousness [21], [22] and auditory hallucinations [23]. The aim of this study was to investigate auditory resting state network connectivity in chronic tinnitus patients.

## Materials and Methods

### Subjects and MRI acquisition

Two independent groups were included. The data of the first healthy control group (group 1) were analyzed in order to select auditory regions of interest (ROIs) subsequently used for auditory independent component selection in group 2. Data from the second group (group 2) were analyzed to compare the auditory resting-state fMRI activity of healthy subjects and tinnitus patients. Healthy volunteers and patients were free of major neurological, neurosurgical or psychiatric history. Head movements were minimized using customized cushions.

*Group 1* included 12 control subjects (4 women; mean age 21 yrs, SD = 3). Resting state BOLD data were acquired on a 3T magnetic resonance scanner (Siemens, Allegra, Germany) with a gradient echo-planar sequence using axial slice orientation (32 slices; voxel size = 3.4×3.4×3 mm^3^; matrix size = 64×64×32; repetition time = 2460 ms, echo time = 40 ms, flip angle = 90°; field of view = 220 mm). A protocol of 350 scans was performed. A T1-weighted MPRAGE sequence was also acquired for registration with functional data on each subject.

*Group 2* included 13 patients (6 women; mean age 52 yrs, SD = 11), with chronic tinnitus present either constantly or intermittently for at least 1 year, and 15 age-matched healthy volunteers (6 women; mean age 51 yrs, SD = 13). Patients with hyperacusis or phonophobia were excluded. Hearing levels were assessed using audiological testing. Pure tones ranging from 250 Hz to 8 kHz were presented to each ear until the threshold of detection was reached. Tinnitus patients were tested to identify the best match to the perceived frequency of their tinnitus. Patients identified the pure tone or white noise from the audiological examination that best matched the center frequency of their tinnitus sensation. Self-reported severity of tinnitus impact was measured using the Tinnitus Handicap Inventory (THI) [24] and the Tinnitus Questionnaire (TQ) [25]. We asked the tinnitus patients to score the tinnitus loudness they experienced during the scanning session directly after the session on a numeric rating scale, ranging from of 0 (none) to 10 (loudest imaginable tinnitus). In group 2, resting state BOLD data were acquired on a 3T magnetic resonance scanner (Siemens, Trio Tim, Germany) with a gradient echo-planar sequence using axial slice orientation (32 slices; voxel size = 3.0×3.0×3.75 mm^3^; matrix size = 64×64×32; repetition time = 2000 ms, echo time = 30 ms, flip angle = 78°; field of view = 192 mm). A protocol of 300 scans lasting 600 seconds was performed. A T1-weighted MPRAGE sequence was also acquired for registration with functional data on each subject.

Written informed consent was obtained from all patients and healthy volunteers. The study was approved by the Ethics Committee of the Faculty of Medicine of the University of Liège.

### Data preprocessing and analysis

fMRI data were preprocessed and analyzed using the “BrainVoyager” software package (Brain Innovation, Maastricht, The Netherlands) and a previously published method [26]. Preprocessing of functional scans included 3D motion correction, linear trend removal, slice scan time correction and filtering out low frequencies of up to 0.005 Hz. The data were spatially smoothed with a Gaussian filter of full width at half maximum value of 8 mm. The functional images from each subject were aligned to the participant's own anatomical scan and warped into the standard anatomical space of Talairach and Tournoux (1988). The spatial transformation was performed in two steps. The first step consisted in rotating the 3-D data set of each subject to be aligned with stereotaxic axes (for this step the location of the anterior commissure, the posterior commissure and two rotation parameters for midsagittal alignment were specified manually). In the second step, the extreme points of the cerebrum were specified. These points together with the anterior commissure and posterior commissure coordinates were then used to scale the 3-D data sets into the dimensions of the standard brain of the Talairach and Tournoux (1988) atlas using a piecewise affine and continuous transformation.

### Auditory component selection

**Figure 1:**
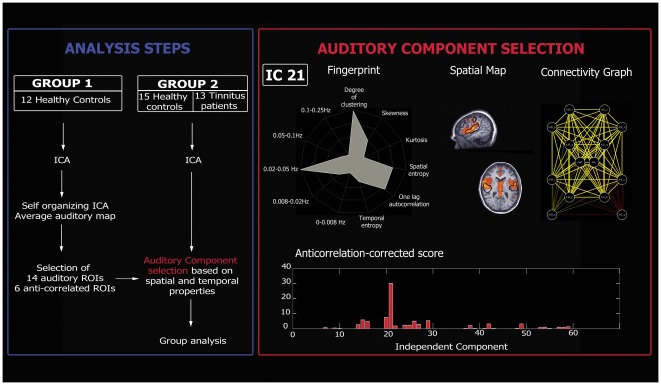
Analysis steps (Blue Box). For the analysis, two independent groups were included. The data of the first group (group 1, healthy controls) were analyzed in order to define auditory regions of interest (ROIs) subsequently used to select the auditory independent component in the second group (group 2, healthy controls and tinnitus patients). Data from group 2 were used to compare the auditory resting-state fMRI activity of healthy subjects and tinnitus patients. **Auditory component selection (Red Box).** The independent component (IC) reflecting the auditory network was selected based on both spatial and temporal properties. *Upper panel (from left to right):* Fingerprint of the selected IC; Spatial map of the selected IC (black contours indicate average auditory map calculated on group 1); Connectivity graph representing significant connectivity edges between the selected ROIs of the auditory network. *Lower panel:* Anticorrelation-corrected score of each graph vs. the corresponding IC number. The component with the highest score will be selected as the auditory network (IC 21 in the present example).

Before investigating spontaneous brain activity, it is necessary to correct the fMRI data for physiological and non-physiological artifacts. To be sure that the further analyzed signal is neurobiologically meaningful and corresponds to the spontaneous brain activity of interest (i.e. the auditory spontaneous activity), we applied independent component analysis. The selection of the components of interest was based on a previously validated selection method which takes advantage of the capability of independent component analysis to decompose the signal in neuronal and artifactual sources while preserving the concept of connectivity in a defined network of ROIs [26]. In order to select the independent component which represent the auditory spontaneous activity, our selection method employed ROIs that were representative regions of previously described auditory resting state network [11], [12], [13], [14]. The ROIs were defined on an average auditory map calculated on a group of twelve independent healthy subjects (group 1). We performed self organizing ICA as implemented in Brain Voyager [27] grouping the 30 independent components of the 12 healthy subjects of group 1 in 30 clusters of spatially similar components. Subsequently, we averaged the maps belonging to the cluster which was selected as auditory by visual inspection. Fourteen ROIs were selected as representative clusters of the Heschl gyrus (Brodman area 41/42), secondary/associative auditory cortices (Brodman area 22) and the insula of our average auditory map *(table S1)*. The ROIs were set initially to a cubic shape 10×10×10 mm^3^, and the center was chosen accordingly to the mean auditory map extracted from group 1 but once the ROI was saved in Brain Voyager only the ROI's voxels belonging to the auditory map end up making the saved ROI. Similarly to the targets ROIs of the auditory component, we then selected six other ROIs representing the most representative regions appearing as anti-correlated regions in the auditory average map calculated on the group 1 of healthy subjects *(table S1)*. These ROIs were used in order to rule out the global signal from the selection. Finally, we picked as auditory component the component that was selected using a compromise between spatial and temporal properties (*figure 1*).

The methodology used, as described by Soddu et al [26], allows building for each independent component a connectivity graph which summarizes the level of connectivity for a defined network of ROIs according to the time behavior described by the correspondent independent component time course. After running ICA with thirty components, we used the corresponding time courses to regress in the BOLD signal in each of the fourteen ROIs. The time courses from each ROI were extracted as the arithmetic mean of the time courses of the voxels belonging to the same ROI. For each component we then obtained fourteen parameter estimates (beta values) indicating the weight of each regressor and the corresponding T-values. In order to build a connectivity graph we drew an edge between each pair of target points with *T>T_th_* with *T_th_* corresponding to 1-p/91 for p = 0.05 with 267 degrees of freedom (Bonferroni correction for multiple comparisons was performed dividing p by the number of possible edges between the thirteen nodes; 14_*_(14-1)/2 = 91). To account for the fact that ICA does not predict the sign of the independent components, the condition *T<-T_th_* was also used. This allowed us to end up with two connectivity graphs for each of the thirty components (1–30 for the condition *T>T_th_* and 31–60 for *T<-T_th_*). We hypothesized that the number of edges *E* for each of the 60 connectivity graphs should be the highest for the auditory component. But given that no regressing out of the global signal was applied, we did not pick the component corresponding to the graph with the largest number of total edges (i.e., the global component could appear as the main source of connectivity). Therefore, we implemented the “anticorrelation-corrected number of edges”. The anticorrelation-corrected number of edges was obtained by multiplying the total number of edges of each graph by a weight “*w*” which measures the anti-correlation of the auditory activity with the set of selected anti-correlated ROIs (w will be around zero for the global component for which all the ROIs are positively correlated). However, to be sure to select a component of neuronal origin one also needs to take into account the temporal properties of the component. To do so, we selected the component with the highest “anticorrelation-corrected score”, built by multiplying the number of anticorrelation-corrected edges by a new weight “*w_F_*” which measures the distance of its fingerprint [28] from the average fingerprint of the auditory component in healthy controls (group 1). The weight *w_F_* is close to 0 for components which have “artefactual” source and close to 1 for components with “neuronal” origin - the latter assumes that in healthy controls ICA was able to fully separate artefactual from neuronal sources *(* *figure 1* *)*.

### Group analysis

Spatial maps were obtained by running a two step analysis. First, the time courses of all components but that of interest (i.e. the independent component selected as auditory) were used to regress out the BOLD signal; the saved residuals represented the BOLD activity which can possibly be explained by the auditory component. Then, by using the time course of the component of interest as a predictor of this residual BOLD activity, beta-values were obtained.

At a second-level analysis, the estimated beta-values entered a multi-subject random effect analysis providing group-level statistical T-maps. Maps were thresholded at a false discovery rate corrected p<0.05. A contrast T-test map was also estimated comparing controls and tinnitus patients. Statistical parametric maps resulting from the voxel wise analysis were considered significant for statistical values that survived a cluster-based correction for multiple comparisons as implemented in Brain Voyager [29] using the “cluster-level statistical threshold estimator” plug-in, which is based on a 3D extension of the randomization procedure described by Forman and colleagues [30]. First, voxel-level threshold was set at t = 2.772 (p = 0.01, uncorrected). After 1000 iterations, the minimum cluster size threshold that yielded a cluster-level false positive rate of 5% was applied to the statistical maps.

## Results

Patients had chronic tinnitus for a mean period of 8 years (SD 9). Tinnitus matched frequencies ranged from 150 Hz to 8 kHz (mean = 4846 Hz, SD = 2276 Hz). Tinnitus Handicap Inventory score [24] varied across patients, from slight to catastrophic (Range: 16–84) as did the Tinnitus Questionnaire (Range: 18–58) [25] *(* *Table 1* *)*. According to the World Health Organization grades of hearing impairment [31], only one tinnitus patient had a grade 1 impairment (slight impairment) all the other had a grade 0 impairment (no impairment). No patients showed profound hearing loss at any frequency (>90 dB above threshold). Four patients didn't exhibit any degree of hearing loss at any of the tested frequencies. The remaining patients exhibited a mild or moderate hearing loss at one or more frequencies (20–40 dB or 40–60 dB above threshold, respectively), and two of these patients demonstrated severe hearing loss in at least one tested frequency (60–90 dB above threshold, on the 4 and 8 kHz).

**Table 1:** Tinnitus Population.

Participant	Sex	Age (years)	Tinnitus Ear	Tinnitus duration (years)	Tinnitus frequency (Hz)	THI/TQ Score	Initial onset related to	Tinnitus loudness during scan (0–10)
***Patient #1***	*F*	*44*	*Right*	*9*	*8000*	*58/35*	*Unknown*	*7*
***Patient #2***	*M*	*47*	*Right*	*33*	*3000*	*38/22*	*Unknown*	*10*
***Patient #3***	*M*	*36*	*Left*	*1.75*	*2500*	*84/58*	*Sudden deafness*	*6.5*
***Patient #4***	*M*	*66*	*Left*	*2*	*4000*	*80/56*	*Earwax extraction*	*8*
***Patient #5***	*M*	*67*	*Left*	*3.75*	*1500*	*30/26*	*Noise trauma*	*5*
***Patient #6***	*M*	*57*	*Bilateral*	*2*	*8000*	*50/52*	*Unknown*	*6.5*
***Patient #7***	*M*	*50*	*Right*	*10*	*6000*	*38/29*	*Stress*	*3*
***Patient #8***	*F*	*60*	*Bilateral*	*>20*	*4000*	*20/20*	*Fatigue*	*3*
***Patient #9***	*F*	*42*	*Right*	*2.4*	*3000*	*40/34*	*Noise trauma*	*2.5*
***Patient #10***	*M*	*33*	*Left*	*3.5*	*8000*	*32/22*	*Unknown*	*4*
***Patient #11***	*F*	*60*	*Bilateral*	*5*	*3000*	*36/21*	*Unknown*	*4.5*
***Patient #12***	*F*	*66*	*Left*	*2*	*6000*	*16/18*	*Hypoacousis*	*4*
***Patient #13***	*F*	*52*	*Left*	*5*	*6000*	*44/22*	*Arnold's neuralgia*	*5*

**Figure 2:**
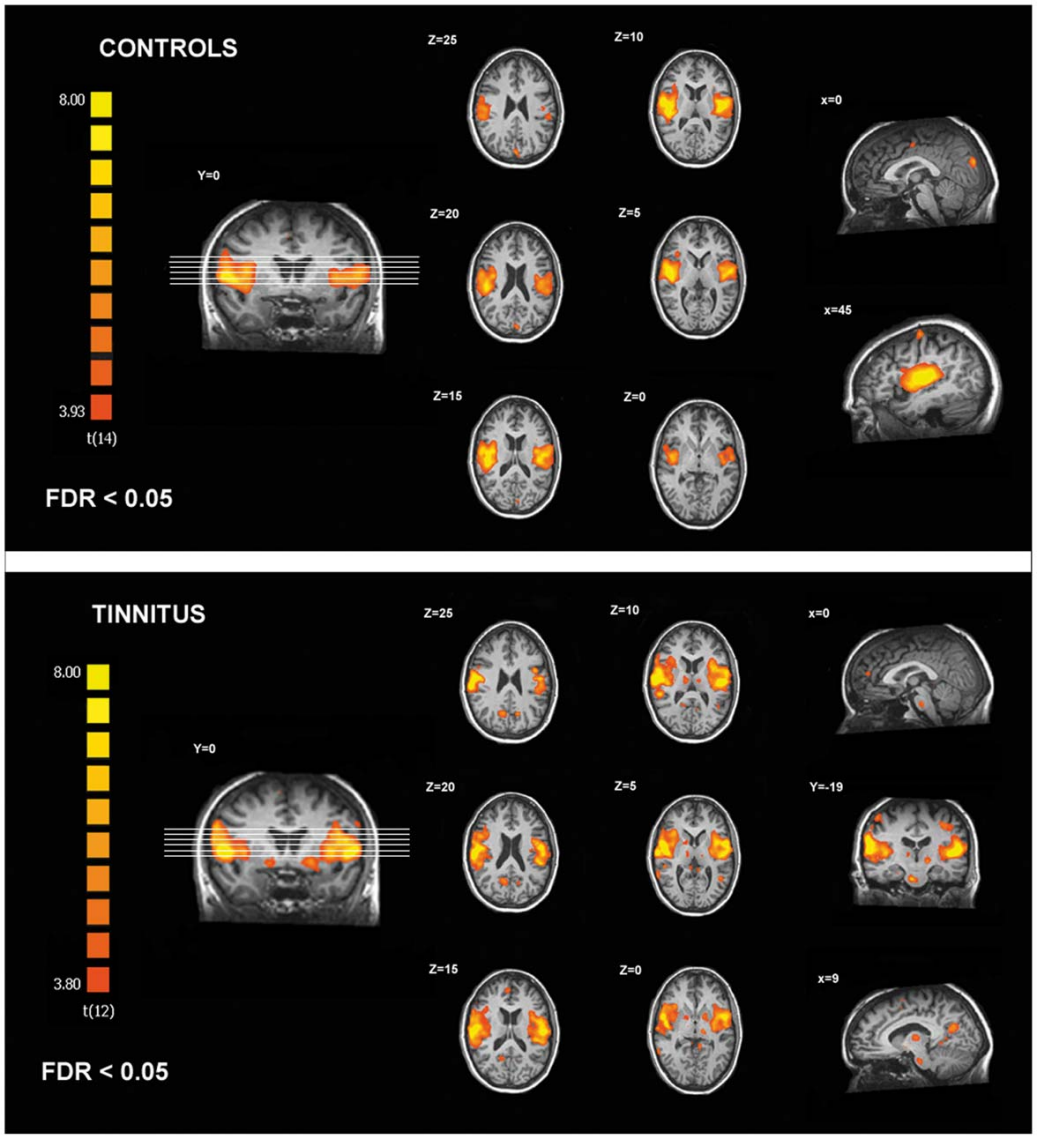
Regions of the auditory resting state network identified in controls and chronic tinnitus patients.

In controls, the identified auditory resting state network encompassed bilateral primary and associative auditory cortices, insula, prefrontal, sensorimotor, anterior cingulate and left occipital cortices (*Table 2* *,* *figure 2*). In tinnitus patients, the identified auditory network encompassed all previously mentioned areas (excluding the anterior cingulate cortex) and included also the brainstem, thalamus, nucleus accumbens (NAc), isthmus of cingulate gyrus, right occipital, parietal and prefrontal cortices (*Table 3* *,* *figure 2*).

**Table 2:** Peak voxels and local maxima of the auditory resting state network identified in controls.

*Brain region (area)*		*x*	*y*	*z*	*t*	*p*
**R**	***Superior & transverse temporal gyrus (41/42/22)***	***49***	***−18***	***11***	***10.81***	***<0.0001***
	*Insula*	*46*	*−12*	*11*	*10.76*	
	*Precentral gyrus (6)*	*58*	*−6*	*11*	*9.59*	
	*Inferior frontal gyrus (45)*	*40*	*21*	*11*	*5.17*	
**L**	***Superior & transverse temporal gyrus (41/42/22)***	***−44***	***−6***	***11***	***10.56***	***<0.0001***
	*Transverse temporal gyrus (42)*	*−59*	*−21*	*17*	*4.93*	
	*Insula*	*−41*	*−18*	*11*	*10.12*	
	*Supramarginal gyrus (40)*	*−47*	*−15*	*14*	*8.43*	
	*Precentral gyrus (6)*	*−53*	*−6*	*8*	*8.50*	
**L**	***Cuneus (18)***	***−6***	***−88***	***37***	***7.35***	***<0.0001***
**R**	***Precentral gyrus (4)***	***45***	***−13***	***58***	***6.11***	***<0.0001***
**R**	***Anterior Cingulate Cortex (24)***	***6***	***−7***	***43***	***5.37***	***<0.0001***

Stereotaxic coordinates are in normalized Talairach space, p values are corrected for multiple comparisons at the whole brain level (FDR<0.05).

**Table 3:** Peak voxels and local maxima of the auditory resting state network identified in the tinnitus patients.

*Brain region (area)*		*x*	*y*	*z*	*t*	*p*
***R***	***Superior & transverse temporal gyrus (41/42/22)***	***62***	***−18***	***23***	***13.97***	***<0.0001***
	*Middle Temporal Gyrus (37)*	*64*	*−48*	*5*	*6.26*	
	*Insula*	*40*	*−18*	*11*	*7.16*	
	*Precentral Gyrus (4)*	*55*	*−9*	*26*	*10.48*	
	*Inferior Frontal Gyrus (44)*	*49*	*9*	*23*	*6.98*	
***L***	***Superior & transverse temporal gyrus (41/42/22)***	***−50***	***−15***	***11***	***11.09***	***<0.0001***
	*Insula*	*−50*	*−33*	*20*	*9.04*	
	*Precentral Gyrus (4)*	*−56*	*6*	*5*	*10.59*	
	*Postcentral Gyrus (3,1,2)*	*−52*	*−9*	*20*	*9.96*	
	*Inferior Frontal Gyrus (44)*	*−50*	*0*	*17*	*7.80*	
	*Basal ganglia/NAc*	*−29*	*−9*	*8*	*7.12*	
***R***	***Cuneus/Precuneus (19/31)***	***9***	***−64***	***25***	***5.88***	***<0.0001***
***L***	***Cuneus/Precuneus (19/31)***	***−15***	***−64***	***25***	***6.20***	***0.0002***
***L***	***Middle occipital gyrus (19)***	***−45***	***−52***	***7***	***6.13***	***<0.0001***
***L***	***Precentral gyrus (4)***	***−33***	***−19***	***46***	***5.27***	***<0.0001***
***R***	***Superior frontal gyrus (6)***	***6***	***5***	***46***	***4.31***	***<0.0001***
***R***	***Prefrontal cortex (10)***	***3***	***47***	***16***	***5.24***	***0.001***
***R***	***Superior parietal cortex (7)***	***54***	***−22***	***52***	***5.61***	***0.0001***
***R***	***Basal ganglia/NAc***	***15***	***−1***	***−5***	***5.61***	***0.0001***
***L***	***Isthmus of Cingulate Gyrus***	***−9***	***−40***	***1***	***5.72***	***0.0003***
***R***	***Thalamus***	***9***	***−13***	***10***	***5.11***	***<0.0001***
***L***	***Thalamus***	***−15***	***−19***	***−2***	***6.44***	***<0.0001***
***R***	***Brainstem***	***6***	***−19***	***−23***	***7.77***	***<0.0001***

Stereotaxic coordinates are in normalized Talairach space, p values are corrected for multiple comparisons at the whole brain level (FDR<0.05).

**Figure 3:**
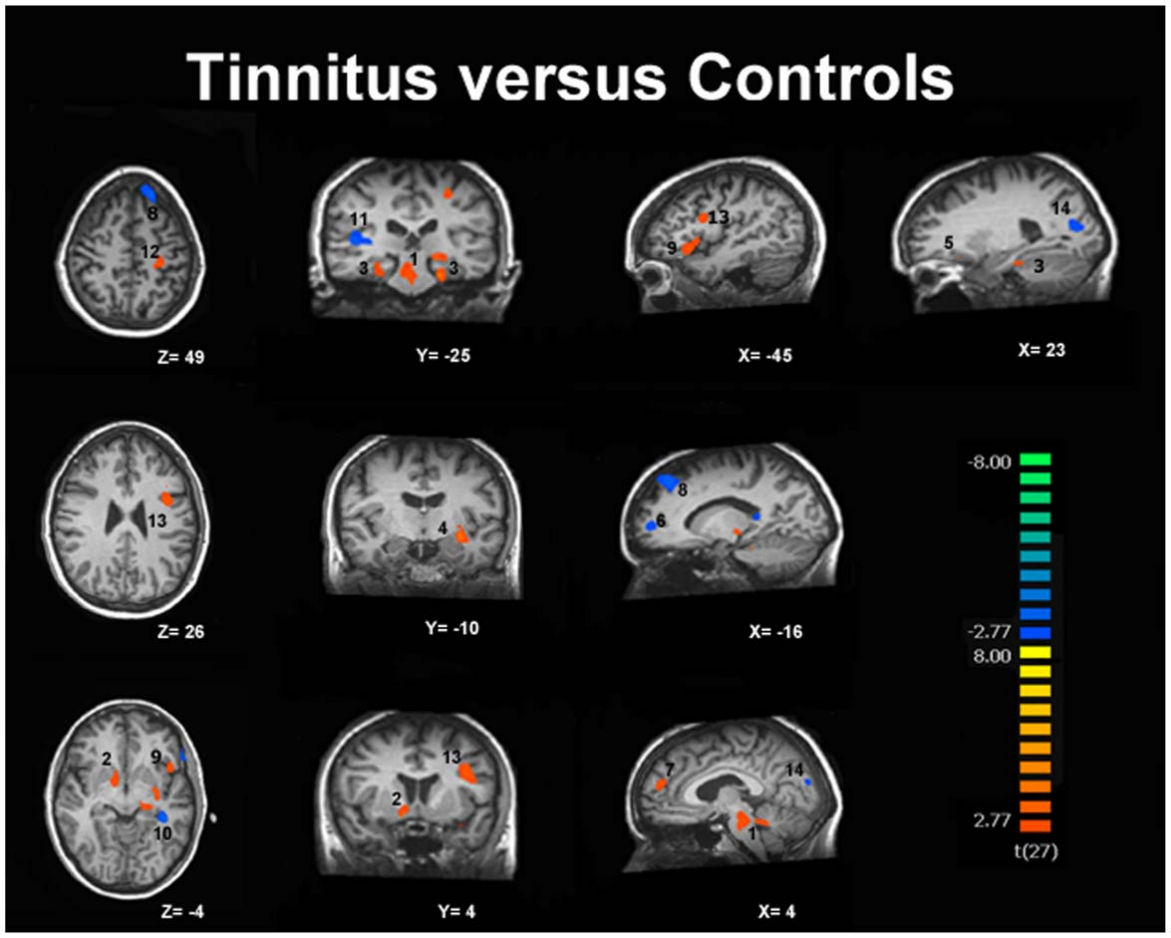
Increased (in red) and decreased (in blue) functional connectivity in the auditory resting-state network in tinnitus. Results are thresholded at cluster level corrected p<0.05. 1- Brainstem/Cerebellum, 2-Basal ganglia/NAc, 3-Parahippocampal gyri, 4-Superior temporal gyrus, 5-Orbitofrontal cortex, 6-Prefrontal cortex, 7-Prefrontal cortex, 8-Superior frontal gyrus, 9-Inferior frontal gyrus, 10-Fusiform gyrus, 11-Superior temporal gyrus, 12-Postcentral gyrus, 13-Precentral gyrus, 14-Cuneus/Precuneus.

Chronic tinnitus patients, as compared to controls, showed increased connectivity in the brainstem, cerebellum, right basal ganglia/NAc, parahippocampal areas, right frontal and parietal areas, left sensorimotor areas and left superior temporal region. Tinnitus patients showed decreased connectivity in right primary auditory cortex, left fusiform gyrus, left frontal and bilateral occipital regions (*Table 4* *,* *figure 3* *, figure S1*).

**Table 4:** Peak voxels of areas showing increased and decreased connectivity in tinnitus as compared to controls.

*Brain region (area)*		*x*	*y*	*z*	*t*	*p*
***INCREASED CONNECTIVITY***						
***L***	*Parahippocampal gyrus*	*−21*	*−28*	*−17*	*4.53*	*0.0001*
***R/L***	*Brainstem/Cerebellum*	*2*	*−21*	*−19*	*4.09*	*0.0004*
***L***	*Precentral gyrus (6)*	*−42*	*2*	*25*	*4.58*	*<0.0001*
***L***	*Superior temporal gyrus*	*−30*	*−10*	*−8*	*4.51*	*0.0001*
***L***	*Inferior frontal gyrus (47)*	*−45*	*14*	*−5*	*3.74*	*0.0009*
***R***	*Basal ganglia/Nucleus accumbens*	*9*	*−1*	*−5*	*4.37*	*0.0002*
***R***	*Prefrontal cortex (10)*	*3*	*50*	*19*	*3.81*	*0.0007*
***L***	*Postcentral gyrus (3,1,2)*	*−33*	*−16*	*43*	*3.69*	*0.001*
***R***	*Parahippocampal gyrus*	*27*	*−25*	*−14*	*3.47*	*0.002*
***R***	*Orbitofrontal cortex (11)*	*30*	*20*	*−11*	*3.83*	*0.0007*
***R***	*Inferior parietal lobe (39)*	*42*	*−52*	*40*	*3.29*	*0.003*
***DECREASED CONNECTIVITY***						
***L***	*Superior frontal gyrus (8)*	*−21*	*38*	*46*	*−4.20*	*0.0003*
***L***	*Fusiform gyrus*	*−39*	*−31*	*−8*	*−4.67*	*<0.0001*
***R***	*Superior temporal gyrus (41)*	*39*	*−28*	*10*	*−4.06*	*0.0004*
***R***	*Occipital cortex (18)*	*21*	*−76*	*16*	*−4.74*	*<0.0001*
***L***	*Occipital cortex (18)*	*−12*	*−85*	*13*	*−3.57*	*0.001*
***L***	*Prefrontal cortex (10)*	*−15*	*53*	*4*	*−4.17*	*0.0003*

Stereotaxic coordinates are in normalized Talairach space (p values are cluster level corrected).

## Discussion

When analyzing spontaneous BOLD fluctuations using fMRI, special care should be taken to disentangle signal changes related to spontaneous neural activity from those related to scanner instability or physiological artifacts due to respiratory, cardiac or motor activity. We here employed the independent component analysis algorithm, decomposing the acquired BOLD signal into different neuronal and non-neuronal components. The selection of the auditory network component was based on a previously published method that allows us to take into account both the spatial and temporal properties of the fMRI signal in order to automatically select the neuronal component of interest in a user-independent manner [26]. The prospectively studied convenience sample of chronic tinnitus patients included subjects with different characteristics regarding tinnitus laterality, frequency and type (pure tone or white noise). Moreover, when looking at the Tinnitus Handicap Inventory and the Tinnitus Questionnaire scores, one could argue that our population was not homogenous regarding the impact of tinnitus on patients' life. This patient inhomogeneity could affect our results mainly by increasing variance and hence decreasing sensitivity. Future studies in larger patient cohorts should aim to correlate specific tinnitus characteristics (such as intensity, localization, type of sound, duration, coping, treatment response) with fMRI BOLD activity.

With the present study we provide evidence for a distributed cerebral network associated with tinnitus. Our data corroborate the hypothesized implication of non-auditory regions in tinnitus physiopathology as proposed by Jastreboff et al [32], [33] (including participation of auditory, limbic, prefrontal areas and autonomic nervous system); Rauschecker et al [34] (suggesting the implication of the NAc and associated paralimbic structures) and De Ridder et al [35] (considering phantom perception -including tinnitus- as a consequence of dysfunction in multiple parallel overlapping dynamic networks -i.e., perception, salience, distress and memory networks-).

The auditory network identified in healthy controls is in line with previous studies using “resting state” fMRI [11], [12], [13], [36]. The observed connectivity impairment in auditory cortex corroborates previous human studies. MEG [37] and EEG studies [38] have demonstrated gamma band activity changes in auditory areas of tinnitus patients and several PET studies have identified primary auditory cortex dysfunction in tinnitus [39], [40], [41], [42].

Our finding of increased connectivity in tinnitus encompassing parahippocampal areas is in accordance with a previous PET study showing increased blood flow in hippocampal areas during tinnitus modified by oral facial movement [40]. Similarly, using EEG, Vanneste et al [8] reported an increase in gamma band frequency in parahippocampal regions and an increase in connectivity between the latter and auditory cortices in tinnitus patients as compared to controls. In fact, primate anatomical studies demonstrated reciprocal connections between parahippocampal regions and associative auditory cortices [43]. Interestingly, De Ridder et al [44], showed that selective amobarbital injections in the anterior choroidal artery (which supplies the amygdalohippocampal region) can suppress tinnitus.

We also found evidence of increased connectivity in the basal ganglia in a region close to the NAc, in line with a fMRI study using auditory stimulations reporting increased activation of the NAc in chronic tinnitus [45]. Rauschecker proposed a tinnitus model in which the NAc and its associated paralimbic networks in the medial prefrontal cortex play an important role. This theory suggests that, under normal circumstances, the tinnitus signal is cancelled out at the level of the thalamus by an inhibitory feedback loop originating in paralimbic structures. If the paralimbic regions are compromised, inhibition of the tinnitus signal at the thalamus gate is lost allowing the signal to reach the auditory cortex where it leads to permanent reorganization and chronic tinnitus [34]. Recently, Larson and colleagues [46] showed that electrical stimulation of the caudate nucleus triggered phantom sounds and modulated tinnitus loudness. These results indicate that the basal ganglia and the NAc might play a key role in tinnitus physiopathology, allowing or not the phantom auditory percept to reach conscious awareness.

The observed tinnitus-related connectivity changes involving the higher-order prefrontal and parietal associative cortices are in line with previous PET [39] and MEG studies [6], [7], [47]. Kleinjung and colleagues showed that tinnitus treatment with repetitive transcranial magnetic stimulation applied on the temporal cortex is enhanced by additional stimulation of the prefrontal cortex [48]. The activation of these regions in tinnitus is consistent with the hypothesis that tinnitus might be associated with an inappropriate allocation of attentional resources, which maintain a sustained state of alertness. Indeed, a multimodal network consisting of temporo-parietal, frontal, and cingulate components is thought to play a key role in identifying and evaluating salient events in the sensory environment, independently of the stimulus modality [49]. Moreover, frontal lobe functioning has also been associated with emotions. An early study, by Beard et al [50], described the effect of frontal leucotomy as a treatment for tinnitus. The effect of frontal lobotomy on tinnitus distress is similar to the effect of lobotomy on pain perception [51]; it was believed to produce asymbolia for pain [52]. Similarly, frontal lobotomy might not alter the tinnitus percept but makes it bearable, dealing with the emotional-behavioral aspect of tinnitus.

Even if considered as the center of motor control, the cerebellum is known to play a role in purely sensory auditory processing [53]. The identified increased functional connectivity in the cerebellum confirms previous PET studies showing increased regional cerebral blood flow in cerebellum when the tinnitus is perceived [39], [54], [55]. At present, few neuroimaging studies in tinnitus reported our observed brainstem involvement. In humans, Lockwood et al [55] used PET to show increased blood flow in the brainstem (supposedly encompassing the cochlear nuclei) correlating with increased tinnitus induced by eye-movements. Finally, the shown connectivity changes within sensorimotor and visual areas could be seen in light of clinical studies showing that tinnitus can be evoked directly or modulated by inputs from somatosensory, somatomotor, and visual-motor systems in a proportion of individuals [56]. These observations give support to the concept that tinnitus could result from, or could be modified by crossmodal neural interactions.

In conclusion, we here provide fMRI evidence for a distributed network of auditory and non-auditory cortical and sub-cortical regions associated with chronic tinnitus. Our results suggest that the tinnitus percept is not only linked to activity in sensory auditory areas but is also associated to connectivity changes in limbic/parahippocampal areas, basal ganglia/NAc, higher-order prefrontal/parietal associative networks, infratentorial brainstem/cerebellar and sensory-motor/visual-motor systems. These results show that there is a modification of cortical and subcortical functional connectivity in tinnitus encompassing attentional, mnemonic and emotional networks. Various tinnitus models suggested the implication of non-auditory regions in tinnitus physiopathology. Our data corroborate these hypotheses and suggest that, even if tinnitus can initially be a perceptual consequence of altered patterns of intrinsic neural activity generated along the central auditory pathway, various regions of the brain seem involved in the persistent awareness of the phenomenon as well as in the development of associated distress leading to disabling chronic tinnitus.

## Supporting Information

Figure S1 **Individual and mean beta-values for each of the cluster found to show significant increased and decreased connectivity in tinnitus as compared to controls.** L Para- Left Parahippocampal gyrus; B/C- Brainstem/Cerebellum; L Pre-Left Precentral gyrus; L STG-Left Superior temporal gyrus; L IFG-Left Inferior frontal gyrus; R BG/NAc-Right Basal ganglia/Nucleus accumbens; R Prefr-Right Prefrontal cortex; L Post-Left Postcentral gyrus; R Para-Right Parahippocampal gyrus; R Orbito-Right Orbitofrontal cortex; R IP-Rigth Inferior parietal lobe; L SFG-Left Superior frontal gyrus; L Fusi-Left Fusiform gyrus; R STG-Rigth Superior temporal gyrus; R Occ-Right Occipital cortex; L Occ- Left Occipital cortex; L Prefr-Left Prefrontal cortex.
(TIF)

Table S1 Regions of interest used for the auditory component selection.
(DOC)
