# Supplementary figures and images for: Auditory Resting-State Network Connectivity in Tinnitus: A Functional MRI Study
Source: PLoS One. 2012 May 4;7(5):e36222. doi: 10.1371/journal.pone.0036222 (PMC3344851; doi:10.1371/journal.pone.0036222)

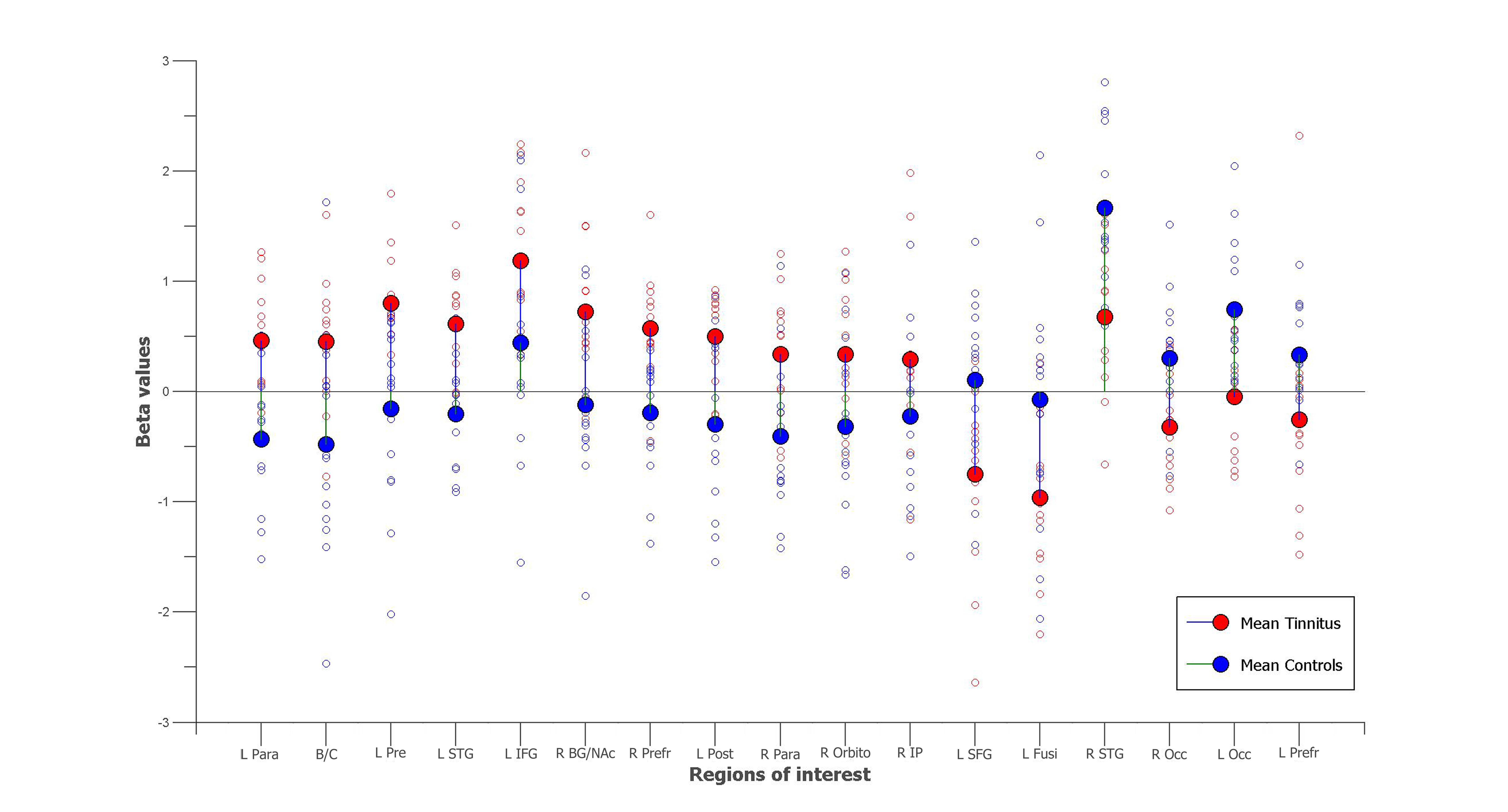

Supplement: Figure S1 — Individual and mean beta-values for each of the cluster found to show significant increased and decreased connectivity in tinnitus as compared to controls. L Para- Left Parahippocampal gyrus; B/C- Brainstem/Cerebellum; L Pre-Left Precentral gyrus; L STG-Left Superior temporal gyrus; L IFG-Left Inferior frontal gyrus; R BG/NAc-Right Basal ganglia/Nucleus accumbens; R Prefr-Right Prefrontal cortex; L Post-Left Postcentral gyrus; R Para-Right Parahippocampal gyrus; R Orbito-Right Orbitofrontal cortex; R IP-Rigth Inferior parietal lobe; L SFG-Left Superior frontal gyrus; L Fusi-Left Fusiform gyrus; R STG-Rigth Superior temporal gyrus; R Occ-Right Occipital cortex; L Occ- Left Occipital cortex; L Prefr-Left Prefrontal cortex. (TIF) [file pone.0036222.s001.tif]
