# Supplementary material for: Auditory Resting-State Network Connectivity in Tinnitus: A Functional MRI Study
Source: PLoS One. 2012 May 4;7(5):e36222. doi: 10.1371/journal.pone.0036222 (PMC3344851; doi:10.1371/journal.pone.0036222)
Supplement: Table S1 — Regions of interest used for the auditory component selection. (DOC) [file pone.0036222.s002.doc]

***Table S1: Regions of interest used for the auditory component selection.***

|  | | ***x*** | ***y*** | ***z*** | ***t*** |
| --- | --- | --- | --- | --- | --- |
| ***AUDITORY REGIONS (area)*** | | | | | |
| *R* | *Transverse temporal gyrus (41)* | *41* | *-23* | *12* | *-3.45* |
| *R* | *Transverse temporal gyrus (41)* | *48* | *-16* | *10* | *-3.52* |
| *R* | *Transverse temporal gyrus (42)* | *55* | *-13* | *6* | *-3.52* |
| *R* | *Superior temporal gyrus (22)* | *54* | *-26* | *11* | *-3.21* |
| *R* | *Superior temporal gyrus (22)* | *55* | *-1* | *5* | *-2.84* |
| *R* | *Superior temporal gyrus (22)* | *54* | *7* | *0* | *-3.24* |
| *R* | *Insula* | *43* | *-6* | *1* | *-3.06* |
| *L* | *Transverse temporal gyrus (41)* | *-43* | *-23* | *12* | *-2.70* |
| *L* | *Transverse temporal gyrus (41)* | *-49* | *-17* | *9.8* | *-3.25* |
| *L* | *Transverse temporal gyrus (42)* | *-55* | *-13* | *6* | *-3.24* |
| *L* | *Superior temporal gyrus (22)* | *-55* | *-25* | *11* | *-3.14* |
| *L* | *Superior temporal gyrus (22)* | *-56* | *-2* | *4* | *-2.99* |
| *L* | *Superior temporal gyrus (22)* | *-55* | *7* | *0* | *-2.77* |
| *L* | *Insula* | *-43* | *-6* | *1* | *-2.74* |
| ***ANTI-CORRELATED REGIONS (area)*** | | | | | |
| *R* | *Precuneus (7)* | *20* | *-78* | *42* | 1.79 |
| *L* | *Precuneus (7)* | *-22* | *-80* | *42* | 1.52 |
| *R* | *Prefrontal cortex (10)* | *18* | *45* | *26* | 0.94 |
| *L* | *Prefrontal cortex (10)* | *-18* | *44* | *26* | 0.71 |
| *R* | *Superior frontal gyrus (6)* | *18* | *13* | *62* | 1.16 |
| *L* | *Superior frontal gyrus (6)* | *-19* | *13* | *62* | 1.25 |
